# Aberrant CXCR4 and β-catenin expression in osteosarcoma correlates with patient survival

**DOI:** 10.3892/ol.2015.3535

**Published:** 2015-07-24

**Authors:** YAO LU, GUO-FENG GUAN, JIE CHEN, BIN HU, CONG SUN, QIONG MA, YAN-HUA WEN, XIU-CHUN QIU, YONG ZHOU

**Affiliations:** 1 Department of Orthopaedic Surgery, Tangdu Hospital, The Fourth Military Medical University, Xi'an, Shaanxi 710038, P.R. China; 2 Department of Haematology, Tangdu Hospital, The Fourth Military Medical University, Xi'an, Shaanxi 710038, P.R. China; 3 Department of Orthopedic Surgery, 537 Hospital of Chinese People's Liberation Army, Baoji, Shaanxi 721006, P.R. China

**Keywords:** C-X-C chemokine receptor type 4, β-catenin, survival, osteosarcoma, immunohistochemistry

## Abstract

To determine the clinical significance of C-X-C chemokine receptor type 4 (CXCR4) and β-catenin in osteosarcoma, their protein expression levels were assessed in 96 osteosarcoma and 20 osteochondroma cases using immunohistochemistry. Additionally, CXCR4 and β-catenin mRNA expression levels were measured in 16 fresh osteosarcoma and 16 adjacent healthy tissue samples using fluorescent reverse transcription-quantitative polymerase chain reaction (RT-qPCR). In the osteosarcoma samples, the positive CXCR4 protein expression rate was significantly higher than the rate in the osteochondroma samples (68.75 vs. 20.00%; P<0.01). Furthermore, β-catenin protein expression was detected in 61.46% of osteosarcoma cases and 25.00% of osteochondroma cases. Similarly, the RT-qPCR data identified increased CXCR4 and β-catenin mRNA expression levels in the osteosarcoma compared with adjacent control tissues. It was determined that CXCR4 (P<0.01) and β-catenin (P<0.05) expression were significantly associated with the clinical Enneking stage, metastasis and survival of osteosarcoma. Furthermore, multivariate analysis identified CXCR4 and β-catenin protein expression levels, as well as clinical stage and metastasis, as significant risk factors for survival in patients with osteosarcoma (P<0.05). In conclusion, the present study determined that CXCR4 and β-catenin are abnormally expressed in osteosarcoma tissues, and, therefore, may be important during osteosarcoma progression.

## Introduction

Osteosarcoma predominantly occurs in children and young adults (1), and is the most common primary malignant bone tumor worldwide (2). With a global incidence of 8.7 cases per million children (age, <20 years) per year, osteosarcoma accounts for ~6% of all childhood cancer (3). Over the previous two decades, advances have been made in the treatment strategies for patients with of osteosarcoma, including in surgery and multimodal chemotherapy. As a consequence, the long-term cure rate for non-metastatic osteosarcoma following surgery has risen from 25 to 60% (4,5). However, despite these advances, the survival rate for patients with osteosarcoma remains low, with novel effective therapeutic strategies required to target this disease. Molecular therapies have been proposed for various types of tumor based on the application of developments in molecular biology. To date, the experimental results have demonstrated good potential for clinical application (6). The C-X-C motif chemokine 12/C-X-C chemokine receptor type 4 (CXCL12/CXCR4) signaling axis is involved in the development of tumors and the metastatic spread of various cancer types (7–9), including osteosarcoma (10). CXCL12 signals through CXCR4, a seven-transmembrane G protein-coupled receptor that is expressed by normal osteoblasts and by malignant cells in osteosarcoma (11,12). Consequently, these proteins have been proposed as potential biomarkers of tumor behavior (13).

The Wnt/β-catenin signaling pathway is important in embryogenesis and organ development (14,15), and has been implicated in the progression and pathogenesis of numerous types of human cancer (16). Dysregulation of Wnt/β-catenin expression is responsible for the invasion and metastasis of osteosarcoma (17), indicating a possible correlation between the CXCR4/CXCL12 axis and Wnt/β-catenin expression in the invasion and metastasis of osteosarcoma. Therefore, the present retrospective study was performed to investigate the *in vivo* expression of CXCR4 and β-catenin in human osteosarcoma, and to analyze the association between the expression of these proteins and clinical prognosis.

## Patients and methods

### 

#### Patients

All patients or their guardians provided written informed consent for participation in the present study. In addition, ethical approval was obtained from the Ethics Committee of the Fourth Military Medical University (Xi'an, China; approval ID: 2013109). Fresh osteosarcoma specimens were obtained from 96 patients who underwent tumor resection at Tangdu Hospital of the Fourth Military Medical University between March 2007 and November 2009. No patients received preoperative chemotherapy or radiotherapy, however, patients with Enneking stage I, II, III or IV disease (18) received postoperative adjuvant chemotherapy [six courses of ifosfamide (2 g/m^2^ for 5 days/course), methotrexate (8 g/m^2^ for 1 day/course) and Adriamycin (50 mg/m^2^ for 1 day/course)]. Of the 96 patients, 44 were female and 52 were male, with a median age of 18 years (range, 8–49 years). A total of 20 osteochondroma specimens were used as the normal controls, including 13 male and 7 female patients with a median age of 20 years (range, 12–56 years). Follow-up care was provided for a minimum of three years. Following resection, formalin-fixed, paraffin-embedded blocks of the osteosarcoma and osteochondroma specimens were retrieved from the Department of Pathology of the Fourth Military Medical University. All samples were evaluated for diagnosis by two similarly experienced pathologists. In addition, 16 pairs of osteosarcoma and adjacent healthy tissue samples were obtained from 16 patients who underwent tumor resection at Tangdu Hospital of the Fourth Military Medical University between July 2013 and December 2013.

#### RNA extraction and reverse transcription-quantitative polymerase chain reaction (RT-qPCR)

Osteosarcoma and adjacent healthy tissue (weight, 25 mg) were harvested, and TRIzol® reagent (Invitrogen Life Technologies, Carlsbad, CA, USA) was used to extract total RNA. First-strand complementary (c)DNA was synthesized using the Avian Myeloblastosis Virus First-Strand cDNA Synthesis kit and oligo(dT) primers (Sangon Biotech Co., Ltd., Shanghai, China), according to the manufacturer's instructions. RT-qPCR was performed using LightCycler®480 software (Roche Diagnostics, Basel, Switzerland) with the SYBR® Green PCR Master Mix (Applied Biosystems, Foster City, CA, USA). β-actin was used as the internal housekeeping gene and relative gene expression was calculated using the cycle threshold (Ct) method (2^−ΔΔCt^). The PCR primers were as follows: Forward, 5′-AATAAAATCTTCCTGCCCACC-3′ and reverse, 5′-CTGTACTTGTCCGTCATGCTTC-3′ for CXCR4; forward, 5′-TGAGCACCTGTTTGCCTGAA-3′ and reverse, 5′-ATGAGCAGCACTCGGACCTT-3′ for β-catenin; and forward, 5′-TAGTTGCGTTACACCCTTTCTTG-3′ and reverse, 5′-TCACCTTCACCGTTCCAGTTT-3′ for β-actin. All experiments were independently performed in triplicate at least three times.

#### Immunohistochemistry

Hematoxylin and eosin-stained osteosarcoma and osteochondroma samples were reviewed by two experienced pathologists to determine the diagnosis and characterize the tumor. The formalin-fixed, paraffin-embedded tissue samples were sectioned at a thickness of 4 µm prior to heating at 60°C in an oven for ≥60 min. The slides were deparaffinized with xylene, hydrated and pretreated with phosphate-buffered saline (PBS; pH 7.4). Subsequently, 3% hydrogen peroxide was used to block endogenous peroxidase activity for 15 min. Slides were incubated overnight with primary antibodies [rabbit polyclonal anti-CXCR4 (cat no. ab2074; Abcam, Cambridge, MA, USA) and rabbit polyclonal anti-β-catenin (cat no. 9562; Cell Signaling Technology, Inc., Boston, MA, USA)] and then with secondary antibodies for 30 min at room temperature. Streptavidin-peroxidase was applied and EnVision™ and universal 3,3′-diaminobenzidine detection kits (Gene Tech Biotechnology Co., Ltd., Shanghai, China) was used with an extra washing step. The slides were counterstained with hematoxylin and mounted. Immunostaining was compared with osteochondroma samples as the normal controls. Negative controls were obtained by substituting the primary antibody with PBS for each protein.

#### Evaluation of immunohistochemistry

CXCR4 and β-catenin immunohistochemistry were examined as previously described (19). Cytoplasmic and membrane immunostaining were distinguished by examining the slides at ×400 magnification using a BX51 microscope (Olympus Corporation, Tokyo, Japan). The extent of immunohistochemical staining was scored as follows: 25% of cells positively stained, 1; 6–50% of cells positively stained, 2; 51–75% of cells positively stained, 3; and 76–100% of cells positively stained, 4. The intensity of CXCR4 and β-catenin expression was scored as negative (0, no staining), weak (1+, only visible at high magnification), moderate (2+, visible at low magnification) and strong (3+, striking at low magnification). For heterogeneous staining, the highest observed level was used for statistical analysis. All cases were scored by two investigators. Multiplying the extent and intensity scores was used to calculate the final immunoreactive score. The IRS of each specimen was categorized into the following four groups: -, 0; +, 1–3; ++, 4–8; and +++, 9–12. Scores of 0–3 and 4–12 were designated as negative and strong expression, respectively. The two investigators reached a consensus on the expression score in all cases.

#### Statistical analysis

A patient was defined as CXCR4- or β-catenin-positive if all respective evaluated samples exhibited strong positive CXCR4 or β-catenin protein expression. Thus, a tumor was negative if all samples from the patient were immunohistochemically negative. All statistical analyses were performed using SPSS software (version 19.0; IBM SPSS, Armonk, NY, USA). To investigate the statistical association between CXCR4 and β-catenin protein expression in the same sample, Pearson's χ^2^ test was used. Correlations between the target protein expression and clinicopathological features were assessed using the χ^2^ test. Furthermore, the Kaplan-Meier product limit method was used to evaluate survival after surgery and multivariate survival analysis was performed using the Cox proportional hazard model. P<0.05 was considered to indicate a statistically significant difference (two-tailed probability).

## Results

### 

#### Expression of CXCR4 and β-catenin in osteosarcoma and osteochondroma

**Figure 1.**
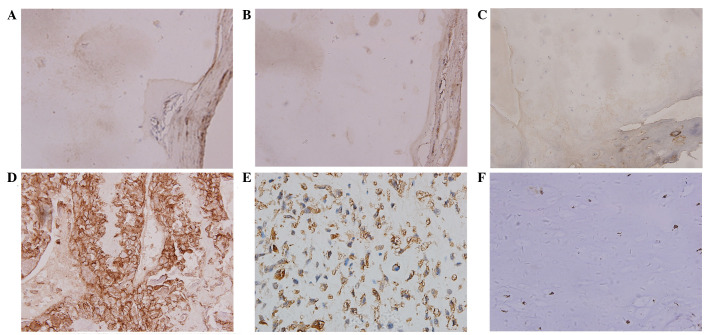
C-X-C chemokine receptor type 4 (CXCR4) and β-catenin expression in human osteosarcoma and osteochondroma tissue (magnification, ×400). Lack of (A) β-catenin and (B) CXCR4 expression, and (C) positive β-catenin expression in osteochondroma. Positive (D) β-catenin and (E and F) CXCR4 expression in osteosarcoma.

**Figure 2.**
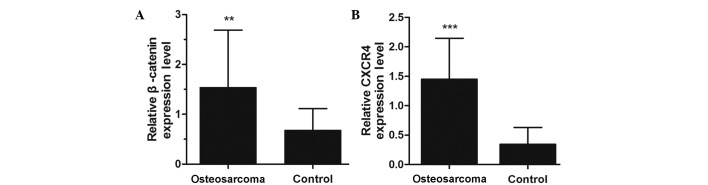
CXCR4 and β-catenin mRNA expression levels in osteosarcoma and healthy control tissue. (A) β-catenin and (B) CXCR4 expression levels were significantly elevated in the osteosarcoma compared with the healthy control tissue samples. **P<0.01 and ***P<0.001 vs. control. CXCR4, C-X-C chemokine receptor type 4.

Immunohistochemistry was used to examine CXCR4 and β-catenin protein expression in samples obtained from osteosarcoma and osteochondroma patients. The different expression levels of the two markers are indicated in Fig. 1. Yellow or brown immunostaining indicated positive CXCR4 expression and was predominantly identified in the cell membrane and cytoplasm. Brown yellow or tan immunostaining indicated positive β-catenin expression and was predominantly identified in the cytoplasm and nucleus. Positive CXCR4 expression was observed in four cases (20.00%) and β-catenin in five cases (25.00%) of osteochondroma (Fig. 1A–C). By contrast, a greater proportion of osteosarcoma samples exhibited CXCR4 and β-catenin expression [68.75% (66/96 cases) and 61.46% (59/96 cases), respectively; Fig. 1D–F; Table I]. The χ^2^ test demonstrated that the expression of these two markers was statistically different in osteosarcoma and osteochondroma (P<0.05; Table I). RT-qPCR data also demonstrated significantly increased levels of β-catenin (P<0.01; Fig. 2A) and CXCR4 (P<0.001; Fig. 2B) mRNA expression in osteosarcoma compared with the adjacent healthy tissue.

#### CXCR4 and β-catenin expression in osteosarcoma patients correlates with clinicopathological features

As indicated in Table II, overall CXCR4 and β-catenin immunostaining were significantly associated with Enneking stage and metastasis (P<0.05). However, the expression of CXCR4 and β-catenin were not significantly associated with gender, age, histological subtype or tumor site.

#### Correlation between CXCR4 and β-catenin protein expression, and patient survival

**Figure 3.**
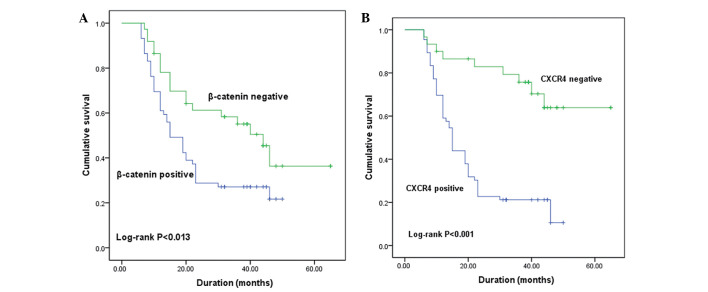
Postoperative Kaplan-Meier survival curves in patients with osteosarcoma according to (A) β-catenin and (B) CXCR4 expression. CXCR4, C-X-C chemokine receptor type 4.

During the follow-up period, 66 patients with osteosarcoma succumbed to the disease. Survival curves correlating immunohistochemical staining patterns with Kaplan-Meier survival are presented in Fig. 3. β-catenin (Fig. 3A) and CXCR4 (Fig. 3B) expression were significant predictors of overall survival (P<0.05; log-rank test). Specifically, survival analysis revealed that a shorter survival time was significantly correlated with patients who demonstrated abnormal (positive) CXCR4 and β-catenin expression. Furthermore, multivariate analysis using the Cox proportional hazard model indicated a significant correlation between overall survival, and CXCR4-positive expression, β-catenin-positive expression, late Enneking stage and the presence of metastases (P=0.000, P=0.018, P=0.000 and P=0.001, respectively). However, age and gender demonstrated no significant association with patient survival (P=0.115 and P=0.457, respectively; Table III).

#### Correlation between CXCR4 and β-catenin protein expression in osteosarcoma

Correlation analysis was performed to determine whether CXCR4 and β-catenin markers were associated with each other in osteosarcoma. The results revealed that β-catenin was positively correlated with CXCR4 expression (r=0.339; P=0.001; Table IV).

## Discussion

Osteosarcoma is the most frequent type of primary bone cancer and typically occurs during childhood or adolescence (2). Osteosarcoma exhibits high local aggressiveness and a high propensity for metastasis, 90% of which is to the lungs (20,21). Furthermore, osteosarcoma metastasis promotes and regulates migratory tumor cells to generate metastatic lesions at distant sites (22), with malignant progression typically resulting in poor prognosis for patients.

A series of complex processes dependent on multiple factors results in the metastasis of a malignant tumor. The well-known role of chemokines in recruiting multiple cell types has, thus far, led the cancer field to focus on the concentration gradients of chemokines and chemokine receptors produced by metastatic sites. Such concentration gradients are known to attract tumor cells to distant locations (23); this evidence is important for explaining why different cancers spread to distinct metastatic sites. Additionally, chemokine receptors appear to be important in the homing of metastatic tumor cells (24,25).

CXCR4 is a major chemokine receptor and is expressed in multiple types of cancer, such as breast and prostate (26,27). Previous studies demonstrated that the application of CXCR4-neutralizing antibodies or small interfering RNA targeting the CXCR4 gene may inhibit metastasis *in vivo* and *in vitro* (28,29). According to the current body of knowledge, CXCR4 is involved in various cancer-related processes, including its development and metastasis (30,31). Therefore, understanding the function of CXCR4 may provide new insights for the development of novel therapeutic strategies for the treatment of cancer.

Inhibition of CXCR4 effectively blocked cancer progression *in vitro* through the traditional Wnt pathway in a previous study (32). CXCR4 was expressed in 20.00% of the osteochondroma samples collected in the current study, whereas CXCR4 was expressed in 68.75% of the osteosarcoma samples. These findings are in agreement with a study by Laverdiere *et al* (33), which demonstrated that CXCR4 expression correlates with metastasis and poor prognosis in patients with osteosarcoma. Additionally, numerous studies identified that the expression of CXCR4 significantly correlates with metastasis in multiple tumor types, including prostate cancer melanoma, breast cancer and rhabdomyosarcoma (34–36). Furthermore, Müller *et al* (27) reported that CXCR4 expression is a key factor in regulating breast cancer metastasis. It was revealed that breast neoplasms expressed high levels of CXCR4, whereas healthy breast tissues expressed low levels.

CXCR4 is a CXCL12 ligand that signals through the CXCL4/CXCR12 axis in a variety of mammals (37). The AMD3100 antagonist is known to block the CXCL4/CXCR12 interaction, resulting in the enhanced mobilization of progenitor cells from bone marrow to peripheral blood (38). Additionally, a number of studies have identified that CXCL12/CXCR4-induced chemotaxis regulates the metastasis of malignant solid tumors (39,40). The current data indicates a correlation between increased CXCR4 expression and a poor prognosis, supporting the possibility of CXCR4 inhibition as a therapeutic target for patients with osteosarcoma. Previously, an inhibiting peptide or a blocking anti-CXCR4 monoclonal antibody were used to specifically inhibit metastasis to the lungs in breast cancer models (27). In addition, non-small cell lung cancer cells with knocked down CXCR4 expression produced larger and more distant tumors compared with wild-type cells, indicating that CXCR4 mediates the metastatic behavior of non-small cell lung cancer (41). Thus, the aforementioned studies provide evidence for the suitability of CXCR4 expression as a prognostic marker and potential therapeutic target in patients with osteosarcoma.

β-catenin is a key protein in the canonical Wnt/β-catenin signaling pathway. Upon activation of the Wnt signaling pathway, β-catenin accumulates in the cytoplasm and is able to translocate to the nucleus, where it engages the DNA-bound T-cell factor transcription factor (42). Previous studies have proposed that abnormal expression of β-catenin may be associated with tumor progression, metastasis and poor prognosis in different cancer types (43,44). Additionally, membrane and cytoplasmic staining indicated that activation of the Wnt/β-catenin signaling pathway is involved in osteosarcoma progression (45).

In the present study, cytoplasmic immunostaining was observed in the majority of osteosarcoma cases (66/96) and the expression of β-catenin was significantly increased in osteosarcoma compared with osteochondroma samples. Furthermore, it was observed that cytoplasmic β-catenin expression was upregulated and membrane-associated β-catenin expression was downregulated in advanced stage tumors. Correlation analysis indicated that aberrant β-catenin expression was significantly associated with metastasis and decreased patient survival. Furthermore, an absence of β-catenin expression significantly correlated with increased patient survival. Aberrant β-catenin and CXCR4 expression were simultaneously observed in 53.1% of the samples. In addition, the current data revealed that CXCR4 and β-catenin mRNA expression were significantly higher in osteosarcoma compared with adjacent healthy tissue. To evaluate these proteins as biomarkers, Spearman correlation coefficient analysis was performed, revealing a significant association between CXCR4 and β-catenin expression.

In conclusion, the present study demonstrated that strong CXCR4 and β-catenin expression were associated with advanced stage disease. Kaplan-Meier survival curves indicated significant differences in clinical prognosis between the β-catenin-positive and β-catenin-negative groups. Furthermore, statistical analysis revealed CXCR4 and β-catenin expression as a predictor of overall survival. Additionally, the present study identified high expression of at least one of the synergistically-regulated mRNAs (CXCR4 or β-catenin) in all osteosarcoma patients. Collectively, the current results indicate that CXCR4 and β-catenin expression may be used as biomarkers to predict prognosis in patients with osteosarcoma and allow for novel therapeutic strategies to be developed.

## Figures and Tables

**Table I.** CXCR4 and β-catenin expression in osteosarcoma and osteochondroma.

		CXCR4, n			β-catenin, n		
					
Group	Cases, n	﹢	-	P-value	﹢	-	P-value
Osteosarcoma	96	66	30		59	37	
Osteochondroma	20	4	16	0.000	5	15	0.006

CXCR4, C-X-C chemokine receptor type 4.

**Table II.** Correlation between CXCR4 and β-catenin expression, and clinicopathological data in patients with osteosarcoma.

	β-catenin, n			CXCR4, n		
				
Variable	-	+	P-value	-	+	P-value
Total	37	59		30	66	
Gender			0.215			0.124
Male	17	35		20	32	
Female	20	24		10	34	
Age, years			0.199			0.497
<20	20	40		17	43	
≥20	17	19		13	23	
Histology			0.200			0.507
Osteoblastic	17	25		12	30	
Chondroblastic	8	10		6	12	
Fibroblastic	8	9		8	9	
Telangiectantic	2	10		3	9	
Mixed	1	6		1	6	
Primary site			0.531			0.189
Femur	21	25		18	28	
Tibia	6	17		4	19	
Humerus	5	9		6	8	
Fibula	2	3		0	5	
Ilium	3	3		1	5	
Other	0	2		1	1	
Distant metastasis			0.001			0.000
Yes	13	42		5	50	
No	24	17		25	16	
Enneking stage			0.047			0.016
I/IIA	9	12		11	10	
IIB	12	8		8	12	
III	16	39		11	44	

CXCR4, C-X-C chemokine receptor type 4.

**Table III.** Multivariate analysis of overall survival in patients with osteosarcoma.

Variable	P-value	RR	95% CI
Gender	0.457	0.827	0.502–1.364
Age	0.115	0.976	0.946–1.006
Metastasis status	0.001	2.487	1.443–4.286
Clinical stage	0.000	1.847	1.319–2.586
CXCR4 expression	0.000	0.301	0.156–0.581
β-catenin expression	0.018	0.304	0.304–0.895

RR, relative risk; CI, confidence interval; CXCR4, C-X-C chemokine receptor type 4.

**Table IV.** Correlation analysis of CXCR4 and β-catenin expression levels.

	β-catenin expression, n	
	
CXCR4 expression	+	-
+	1	5
-	8	22

R=0.339; P=0.001. CXCR4, C-X-C chemokine receptor type 4.
